# MetaPathways v2.5: quantitative functional, taxonomic and usability improvements

**DOI:** 10.1093/bioinformatics/btv361

**Published:** 2015-06-15

**Authors:** Kishori M. Konwar, Niels W. Hanson, Maya P. Bhatia, Dongjae Kim, Shang-Ju Wu, Aria S. Hahn, Connor Morgan-Lang, Hiu Kan Cheung, Steven J. Hallam

**Affiliations:** ^1^Department of Microbiology & Immunology, University of British Columbia, 2350 Health Sciences Mall, Vancouver, BC, Canada,; ^2^Graduate Program in Bioinformatics, University of British Columbia, Genome Sciences Centre, 100-570 West 7th Avenue, Vancouver, BC, Canada and; ^3^Department of Computer Science, University of British Columbia, 2366 Main Mall, Vancouver, BC, Canada

## Abstract

**Summary:** Next-generation sequencing is producing vast amounts of sequence information from natural and engineered ecosystems. Although this data deluge has an enormous potential to transform our lives, knowledge creation and translation need software applications that scale with increasing data processing and analysis requirements. Here, we present improvements to MetaPathways, an annotation and analysis pipeline for environmental sequence information that expedites this transformation. We specifically address pathway prediction hazards through integration of a weighted taxonomic distance and enable quantitative comparison of assembled annotations through a normalized read-mapping measure. Additionally, we improve LAST homology searches through BLAST-equivalent E-values and output formats that are natively compatible with prevailing software applications. Finally, an updated graphical user interface allows for keyword annotation query and projection onto user-defined functional gene hierarchies, including the Carbohydrate-Active Enzyme database.

**Availability and implementation:** MetaPathways v2.5 is available on GitHub: http://github.com/hallamlab/metapathways2.

**Contact:** shallam@mail.ubc.ca

**Supplementary information:** Supplementary data are available at *Bioinformatics* online.

## 1 Introduction

Since the publication of MetaPathways (Konwar *et* *al.*, 2013), a modular annotation and analysis pipeline that enables construction of environmental pathway/genome databases using Pathway Tools (Karp *et* *al.*, 2002b, 2010) and MetaCyc (Caspi *et al.*, 2012; Karp *et* *al.*, 2000, 2002a), there have been improvements to the software via the Knowledge Engine data structure, a graphical user interface (GUI) for data management and browsing and a master–worker model for task distribution on grids and clouds (Hanson *et* *al.*, 2014b). Version 2.5 features faster and more accurate quantitative functional and taxonomic inference. Inspired by the pathway-centric analysis of the Hawaii-Ocean Time-series (Hanson *et* *al.*, 2014a), a weighted taxonomic distance (WTD) has been integrated to detect taxonomic divergence of predicted MetaCyc pathways. Next, because it is difficult to determine relative open reading frame (ORF) abundance in assembled datasets, we adopt reads per kilobase per million mapped (RPKM) to provide a quantitative measure of sequence-coverage on a per-ORF basis (Patil *et* *al.*, 2011). Additionally, the LAST code has been modified to calculate BLAST-equivalent Bit-score and E-value statistics (Altschul *et al.*, 1990; Kiełbasa *et al.*, 2011), producing output files compatible with prevailing software applications, including the MetaGenome ANalyser (Huson *et* *al.*, 2007). Finally, query and projection features have been enhanced with keyword-based searches, with support for Carbohydrate-Active EnZymes database entries (Cantarel *et* *al.*, 2009).

## 2 Methods

Here, we describe MetaPathways v2.5 improvements in more detail.

### 2.1 Weighted taxonomic distance

MetaPathways runs the PathoLogic algorithm without taxonomic pruning, but this omission enables prediction of MetaCyc pathways outside their expected taxonomic range. WTD serves as a measure of predicted pathway taxonomic divergence between observed RefSeq taxonomy and its expected taxonomic range (Hanson *et* *al.*, 2014a). Briefly, for each predicted pathway *P*, WTD *D* is calculated on the connecting path $P(x_{\text{exp}},x_{\text{obs}})$ between *x*_obs_, the lowest common ancestor of observed annotations, and each member of its expected taxonomic range *x*_exp_,

(1) $$D(x_{\text{exp}},x_{\text{exp}})=\sum_{e_{\text{a,b}}\in E_{P(x_{\text{exp}},x_{\text{obs}})}}\frac{1}{2^{d(a)}}$$

where *e*_a,b_ is an edge between nodes *a* and *b* on the connecting path $E_{P(x_{\text{exp}},\;x_{\text{obs}})}$, and *d*(*a*) is the depth of node *a.* (For complete algorithm details and motivation, see Online Methods and Supplementary Note S2 of *Metabolic pathways for the whole community* (Hanson *et al.*, 2014a)).

### 2.2 Reads per kilobase per million mapped

Functional analysis of *de novo* assembled environmental sequence information is impeded by the lack of quantitative ORF annotations. ORF counts are affected by both sequencing depth and ORF length, longer ORFs naturally encompass more reads, making quantitative comparisons between samples difficult. To resolve this, we have implemented a bwa-based version of the RPKM (Li and Durbin, 2010). Intuitively RPKM is a simple proportion of the number of reads mapped to a sequence section, normalized for sequencing depth and ORF length:

(2) $$\text{RPKM}=\frac{\frac{\text{Reads}\;\text{Mapped}\;\text{to}\;\text{ORF}}{\text{ORF}\;\text{Length}\;\text{(kb)}}}{\frac{\text{Reads}\;\text{Mapped}\;\text{to}\;\text{Sample}}{{\text{10}}^{\text{6}}}}$$

### 2.3 LAST bit-score and E-value

Although both LAST and BLAST are dynamic programming seed-and-extend approximations to the Smith Waterman algorithm (Altschul and Erickson, 1986; Smith and Waterman, 1981), in practice, LAST’s adaptive-seed lengths and simpler code base is 20- to 100-times faster, more accurate and portable. However, LAST adoption has lagged due to the absence of BLAST-like output format and statistics. We modified the LAST code to produce the compatible Bit-score and E-value calculations.

## 3 Results

**Fig. 1.**
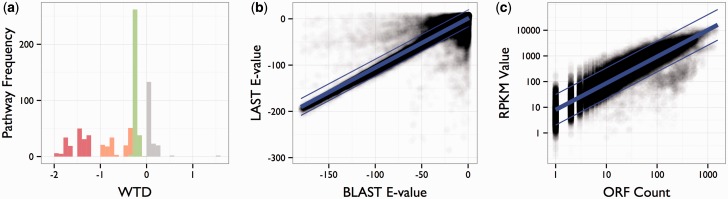
Quantitative functional and taxonomic improvements. **(a)** WTD provides a measure of taxonomic agreement between observed RefSeq Lowest common ancestor (LCA) taxonomy and the expected taxonomic range of predicted MetaCyc pathways, separated into the ‘High’ (Red), ‘Medium’ (Orange) and ‘Low’ (Green) taxonomic hazard classes based on negative quartile order statistics. Positive distances represent taxa found within a pathways expected taxonomic range and so have a hazard class of ‘None’ (Grey). **(b)** The LAST and BLAST homology search algorithms are highly correlated in terms of E-value ($R^{2}=0.887$, *P* < 0.01). **(c)** ORF Count and the RPKM measure show a linear relationship (*R*^2 ^= 0.816, *P* < 0.01). Ninety percent of prediction intervals, displayed as a pair of thin blue lines about the fitted line, capture ∼96.7 and 91.3% of observed points in **(**b**)** and **(**c**)**, respectively. Analysis code can be found in the Supplementary information

We benchmarked the implemented improvements described earlier using Illumina-sequenced marine metagenomic samples. (Joint Genome Institute: ‘Marine microbial communities from Expanding Oxygen minimum zones project’ (JGI Project IDs: 4093112, 4093113, 4093125, 4093127–4093132, 4093144–4093149, 4096364–4096371, 4096373, 4096375, 4096377–4096379, 4096381–4096383, 4096385–4096387, 4096389–4096396, 4096398–4096406 and 4096409–4096453)). The WTD distribution can be used as an informative tool to place pathways into different taxonomic hazard classes based on their order statistics (Fig. 1a). Protein annotations of BLAST and LAST are highly correlated in terms of E-value (Fig. 1b), suggesting roughly equivalent results, but with LAST being significantly faster. Although there is a positive correlation between RPKM score and ORF count, variance about the regression line indicates RPKM makes a correction in many instances (Fig. 1c).

## 4 Conclusions

MetaPathways v2.5 now addresses quantitative functional and pathway prediction hazards based on WTD and RPKM calculations, provides performant LAST output equivalent with BLAST, and more flexible annotation subsetting and projection via GUI keyword searches. These improvements enable improved large-scale comparative analysis of next-generation environmental sequence information.

## Supplementary Material

Supplementary Data
