# Supplementary material for: MetaPathways v2.5: quantitative functional, taxonomic and usability improvements
Source: Bioinformatics. 2015 Jun 15;31(20):3345–7. doi: 10.1093/bioinformatics/btv361 (PMC4595896; doi:10.1093/bioinformatics/btv361)
Supplement: Supplementary Data [file supp_31_20_3345__index.html]

MetaPathways v2.5: Quantitative functional, taxonomic, and usability improvements — MetaPathways v2.5: quantitative functional, taxonomic and usability improvements — MetaPathways v2.5: quantitative functional, taxonomic and usability improvements — Supplementary Data 

# MetaPathways v2.5: quantitative functional, taxonomic and usability improvements

## Supplementary Data

files

- Supplementary Data - gz file
- Supplementary Data - gz file
- Supplementary Data - gz file
- Supplementary Data - gz file
- Supplementary Data - gz file
- Supplementary Data - zip file
