# Supplementary material for: MetaPathways v2.5: quantitative functional, taxonomic and usability improvements
Source: Bioinformatics. 2015 Jun 15;31(20):3345–7. doi: 10.1093/bioinformatics/btv361 (PMC4595896; doi:10.1093/bioinformatics/btv361)
Supplement: Supplementary Data [file supp_btv361_mp_2_5_supplement_html.zip › mp_2_5_supplement.html]

MetaPathways v2.5: Supplementary Material


# MetaPathways v2.5: Supplementary Material

#### *Kishori M. Konwar, Niels W. Hanson, Maya P. Bhatia, Dongjae Kim, Shang-Ju Wu, Aria S. Hahn, Connor Morgan-Lang, Hiu Kan Cheung, and Steven J. Hallam*

#### *December 19, 2014*

- 1 Load Required R Packages and Data
- 2 WTD Hazard Calculation
- 3 LAST vs. BLAST E-value Comparison
- 4 ORF Counts vs. RPKM

The MetaPathways v2.5 release is available from GitHub with binaries compatible for various Linux and Mac OSX Operating Systems:

http://github.com/hallamlab/metapathways2/releases

# 1 Load Required R Packages and Data

Data files can be found here.

- Pathways
  - 01\_jgi\_4093112\_combined\_unique.pwy.txt.gz: MetaCyc Pathway predictions and WTD calculations
- BLAST and LAST outputs re
  - 02\_4093112\_combined\_unique.qced.faa.cog.last.common.txt.gz: Standard tabular BLAST results.
  - 03\_4093112\_combined\_unique.qced.faa.cog.blast.common.txt.gz: Modified LAST output with E-value.
- RPKM
  - 04\_rpkm\_master\_table\_ORF\_count.txt.gz: Master table of open reading frame (ORF) counts for all predicted MetaCyc Pathways.
  - 05\_rpkm\_master\_table\_rpkm.txt.gz: Master table Reads per kilobase per-million mapped (RPKM) values for all predicted MetaCyc Pathways.

We are using ggplot2, reshape2, and plyr R packages for plotting and data manipulation.

- Load libraries.

```
library(ggplot2)
library(reshape2)
library(plyr)
```

- Load data

```
pwys <- read.table("data/01_jgi_4093112_combined_unique.pwy.txt.gz", header=T, sep="\t")
blast <- read.table("data/02_4093112_combined_unique.qced.faa.cog.blast.common.txt.gz", comment.char="#", sep="\t", header=FALSE)
last <- read.table("data/03_4093112_combined_unique.qced.faa.cog.last.common.txt.gz", comment.char="#", sep="\t", header=FALSE)          
ORFs <- read.table("data/04_rpkm_master_table_ORF_count.txt.gz", sep="\t", header=T)
RPKMs <- read.delim("data/05_rpkm_master_table_rpkm.txt.gz", sep="\t", header=T)
```

# 2 WTD Hazard Calculation

Here, we’ll load the `.pwy.txt` file found found in the `<sample>/results/pgdb/` folder of a MetaPathways output, which now includes WTD calculated for each predicted MetaCyc pathway. We’ll classify each predicted pathways based on the negative quartiles of the sample, and plot a histogram of the classification.

- Classify by order-statistics

```
# set all to default hazard class
pwys$hazard_class = "None"

# calculate summary statistics of negative WTD scores and set hazard classes
wtd_stats <- summary(pwys$WTD[pwys$WTD < 0])
pwys$hazard_class[pwys$WTD > wtd_stats["Median"]] = "Low"
pwys$hazard_class[pwys$WTD > wtd_stats["1st Qu."] & pwys$WTD <= wtd_stats["Median"]] = "Medium"
pwys$hazard_class[pwys$WTD <= wtd_stats["1st Qu."]] = "High"
pwys$hazard_class[pwys$WTD >= 0] = "None"

# factor new levels
pwys$hazard_class <- factor(pwys$hazard_class, levels=c("None", "Low", "Medium", "High"))
```

- Plot distribution

```
# create Figure 1a
p1 <- ggplot(pwys, aes(x=WTD)) + geom_histogram(aes(fill=hazard_class), binwidth=0.1)
p1 <- p1 + scale_fill_manual(name="Hazard Class",
                             values=c("#CCCCCC", "#AFE591", "#FAA982", "#DF737D")) 
p1 <- p1 + theme_bw(base_family="Gill Sans")
p1 <- p1 + ylab("Pathway Frequency")
p1 <- p1 + ggtitle("a")
p1 <- p1 + theme(plot.title=element_text(size=30, vjust = 2),
                 axis.title.x = element_text(size=14, vjust=-0.3),
                 axis.title.y = element_text(size=14, vjust=1),
                 legend.position="none")
p1
```

# 3 LAST vs. BLAST E-value Comparison

We modified the source code of LAST, and implemented BLAST-equivalent E-values and file output. Here we compare the E-values of BLAST and LAST on a common sample, and fit a linear model to show their correlation.

- Prepare data

```
last$algo = "LAST"
blast$algo = "BLAST"

df <- rbind(last[-13], blast)
df <- cbind(paste(df$V1, df$V2, sep = "_"),df)

header <- c("hit", "query", "subject", "identity", "al_length", "missmatch", "gap_open", 
            "query_start", "query_end", "subject_start", "subject_end",
            "eval", "bit_score", "algo")

colnames(df) <- header
df.m <- melt(df)
```

- Fit Linear Model

```
df.m.eval <- dcast(subset(df.m, variable=="eval"), hit~algo, max)
df.m.eval$BLAST <- log(df.m.eval$BLAST) / log(10)
df.m.eval$LAST <- log(df.m.eval$LAST) / log(10)
df.m.eval[df.m.eval == "-Inf"] = NA
df.m.eval <- df.m.eval[complete.cases(df.m.eval),]
eval_fit <- lm(LAST ~ BLAST, data=df.m.eval)
summary(eval_fit)
```

```
## 
## Call:
## lm(formula = LAST ~ BLAST, data = df.m.eval)
## 
## Residuals:
##      Min       1Q   Median       3Q      Max 
## -269.215   -1.724    0.071    2.315  194.728 
## 
## Coefficients:
##              Estimate Std. Error t value Pr(>|t|)    
## (Intercept) 0.8717725  0.0226312   38.52   <2e-16 ***
## BLAST       1.0639488  0.0005121 2077.70   <2e-16 ***
## ---
## Signif. codes:  0 '***' 0.001 '**' 0.01 '*' 0.05 '.' 0.1 ' ' 1
## 
## Residual standard error: 11.06 on 548061 degrees of freedom
## Multiple R-squared:  0.8873, Adjusted R-squared:  0.8873 
## F-statistic: 4.317e+06 on 1 and 548061 DF,  p-value: < 2.2e-16
```

- Add 90% prediction intervals to dataframe

```
pred_alpha=0.90
pred_interval <- predict(eval_fit, df.m.eval, interval="prediction", level=pred_alpha)
df.m.eval$lwr <- pred_interval[,"lwr"]
df.m.eval$upr <- pred_interval[,"upr"]

within <- df.m.eval$LAST > df.m.eval$lwr & df.m.eval$LAST < df.m.eval$upr
df.m.eval$within <- within
cap_precent <- sum(within) * 100 / length(within)
cap_percent <- round(cap_precent, 3)

df.m.eval.pred <- melt(df.m.eval[,c("BLAST","lwr","upr")], id.vars = c("BLAST"))
```

- 90% prediction intervals capture 96.707% of the observed data
- Plot scatterplot with fit 90% prediction interals as thin lines

```
# create Figure 1b
df.m.eval.plot <- df.m.eval
p2 <- ggplot(df.m.eval.plot, aes(x=BLAST, y=LAST))
p2 <- p2 + geom_point(size=3, alpha=0.005)
# p2 <- p2 + scale_color_manual(values=c("#4959FE", "#000000")) 
p2 <- p2 + theme_bw(base_family="Gill Sans")
p2 <- p2 + stat_smooth(formula="y ~ x", method="lm", size=2, color="#464BFF")
p2 <- p2 + xlab("BLAST E-value") + ylab("LAST E-value")
p2 <- p2 + ggtitle("b")
p2 <- p2 + theme(plot.title=element_text(size=30, vjust = 2),
                 axis.title.x = element_text(size=14, vjust=-0.3),
                 axis.title.y = element_text(size=14, vjust=1))
p2 <- p2 + theme(legend.position="none")
p2 <- p2 + geom_line(data=df.m.eval.pred, aes(x=BLAST, y=value, group=variable), color="#464BFF", size=0.5)
p2
```

# 4 ORF Counts vs. RPKM

Finally, we’ll compare ORF counts to the RPKM statistics for predicted MetaCyc pathways accross 91 metagenomes.

- Prepare Data Frame of ORF and RPKM counts
- Remove instances where there were both counts are zero

```
ORFs$measure <- "ORFs"
RPKMs$measure <- "RPKMs"

ORFs.m <- melt(ORFs, id.vars=c("pwy_short", "pwy_long", "measure"))
RPKMs.m <- melt(RPKMs, id.vars=c("pwy_short", "pwy_long", "measure"))
df <- rbind(ORFs.m, RPKMs.m)
df.measure <- dcast(df, pwy_short*variable~measure)
df.measure$ORFs <- as.numeric(df.measure$ORFs)

# remove those where both zero
df.measure.sub <- subset(df.measure, ORFs != 0 & RPKMs != 0)
```

- Fit Linear Model between ORF and RPKM counts

```
set.seed(39294)
samp_size <- round(nrow(df.measure.sub)/2)
samp <- sample(1:nrow(df.measure.sub), samp_size)

rpkm_fit <-  lm(I(log(RPKMs))~I(log(ORFs)), data=df.measure.sub)
summary(rpkm_fit)
```

```
## 
## Call:
## lm(formula = I(log(RPKMs)) ~ I(log(ORFs)), data = df.measure.sub)
## 
## Residuals:
##     Min      1Q  Median      3Q     Max 
## -7.4569 -0.4274  0.0247  0.4858  4.4134 
## 
## Coefficients:
##              Estimate Std. Error t value Pr(>|t|)    
## (Intercept)  2.076642   0.006976   297.7   <2e-16 ***
## I(log(ORFs)) 1.035903   0.001943   533.2   <2e-16 ***
## ---
## Signif. codes:  0 '***' 0.001 '**' 0.01 '*' 0.05 '.' 0.1 ' ' 1
## 
## Residual standard error: 0.8348 on 64083 degrees of freedom
## Multiple R-squared:  0.8161, Adjusted R-squared:  0.816 
## F-statistic: 2.843e+05 on 1 and 64083 DF,  p-value: < 2.2e-16
```

- Add 90% prediction intervals to dataframe

```
rpkm_fit <- lm(I(log(RPKMs))~I(log(ORFs)), data=df.measure.sub)
pred_interval <- predict(rpkm_fit, df.measure.sub, interval="prediction", level = pred_alpha)
df.measure.sub$lwr <- exp(pred_interval[,"lwr"])
df.measure.sub$upr <- exp(pred_interval[,"upr"])

within <- df.measure.sub$RPKMs > df.measure.sub$lwr & df.measure.sub$RPKMs < df.measure.sub$upr
cap_precent <- sum(within) * 100 / length(within)
cap_percent <- round(cap_precent, 3)
df.measure.sub$within <- within

df.measure.sub.pred <- melt(df.measure.sub[,c("ORFs","lwr","upr")], id.vars = c("ORFs"))
```

- 90% prediction intervals capture 91.318% of the observed data
- Plot scatterplot with fit and 90% prediction interals as thin lines

```
# create Figure 1c
p3 <- ggplot(df.measure.sub, aes(ORFs, RPKMs))
#p3 <- p3 + geom_point(size=3, alpha=0.01, aes(color=within)) 
#p3 <- p3 + scale_color_manual(values=c("#4959FE", "#000000")) 
p3 <- p3 + geom_point(size=3, alpha=0.005)
p3 <- p3 + scale_color_manual(values="#000000")
p3 <- p3 + scale_x_log10(breaks=c(1,10,100,1000))
p3 <- p3 + scale_y_log10(breaks=c(1,10,100,1000,10000)) 
p3 <- p3 + stat_smooth(method="lm", formula="y~x", size=2, color="#464BFF") 
p3 <- p3 + theme_bw(base_family="Gill Sans")
p3 <- p3 + ylab("RPKM Value")
p3 <- p3 + xlab("ORF Count")
p3 <- p3 + ggtitle("c")
p3 <- p3 + theme(plot.title=element_text(size=30, vjust = 2),
                 axis.title.x = element_text(size=14, vjust=-0.3),
                 axis.title.y = element_text(size=14, vjust=1))
p3 <- p3 + theme(legend.position="none")
p3 <- p3 + geom_line(data=df.measure.sub.pred, aes(x=ORFs, y=value, group=variable), color="#464BFF", size=0.5)
p3
```

- Put all three together for figure using Winston Chang’s `multiplot()` function
- Plot figures together

```
multiplot(p1, p2, p3, cols=3)
```

Any questions or comments, please contact Dr. Steven J. Hallam (shallam@mail.ubc.ca).
